# Exploring the enablers and barriers to social prescribing for people living with long-term neurological conditions: a focus group investigation

**DOI:** 10.1186/s12913-021-07213-6

**Published:** 2021-11-13

**Authors:** Suzanne Simpson, Moira Furlong, Clarissa Giebel

**Affiliations:** 1 The Walton Centre NHS Foundation Trust, Lower Lane, Fazakerely, Liverpool, Merseyside L9 7LJ UK; 2 NIHR ARC NWC, Liverpool, UK; 3 MND Association, Northampton, UK; 4 Department of Primary Care & Mental Health, University of Liverpool, Liverpool, UK

**Keywords:** Neurological conditions, Social prescribing, Enablers, Barriers, Link workers, Wellbeing, Occupational therapy

## Abstract

**Background:**

People living with Long Term Neurological Conditions (LTNCs) value peer support and social activities. Psychological support and wellbeing enables them to manage their condition. Social prescribing is a formal process of referring patients to a link worker to co-design a plan to improve their health and wellbeing. Intervention involves supporting participation in activities based within the individual’s local community. This study aimed to explore the barriers and enablers to accessing social prescribing for people living with LTNCs (plwLTNCs).

**Methods:**

A total of four focus groups were carried out with 17 participants, including different neurological conditions such as multiple sclerosis, Fragile X Syndrome, epilepsy, and traumatic brain injury. Two participants were family carers and supported people living with epilepsy and motor neurone disease. Findings were analysed using thematic analysis.

**Results:**

Five themes were identified: (1) Lack of knowledge; (2) Service provision difficulties; (3) Benefits of social prescribing activities; (4) Physical barriers and (5) Psychological barriers. There was a lack of knowledge about social prescribing and what it actually was. Participants anticipated service provision difficulties relating to funding, link workers need for knowledge of LTNC’s and for activities to be varied and individualised. The potential benefits of social prescribing activities were recognised across the groups especially its potential to tackle loneliness and to offer plwLTNC’s purpose. Participants highlighted a number of physical barriers such as transport and accessibility; and psychological barriers such as anxiety and stigma.

**Conclusion:**

Social prescribing aims to address the health inequalities of those living with long-term conditions, however currently it is likely to exclude plwLTNCs. Recommendations for practice and future research are made.

**Supplementary Information:**

The online version contains supplementary material available at 10.1186/s12913-021-07213-6.

## Introduction

There are an estimated 14.7 million neurological cases in England, equating to at least 1 in 6 people living with one or more neurological condition [1]. A Long-Term Neurological Condition (LTNC) results from injury, damage to, or disease of the nervous system (brain, spinal cord, peripheral or autonomic nervous system) [2]. Neurological conditions make up 20% of all long-term conditions and include a wide range of illnesses [2]. In 2018 the Neurological Alliance GP survey revealed that 19% of patients living with a neurological condition had had an unplanned admission to hospital in a period of 12 months, which is twice the rate for all people with a long-term condition (9.8%) [3]. People living with neurological conditions have the lowest health related quality of life of any long-term condition and deaths are 35% more likely to be premature [4]. Data produced by NHS RightCare (2019) suggests there is a substantial financial savings opportunity in relation to reducing emergency admissions and bed days for people living with neurological conditions [5].

Research into quality of life in these patient groups, such as the Trajectories of Outcome in Neurological Condition (TONiC), has found that patients emphasise the importance of psychological support and wellbeing in helping them manage their condition [6]. The Neurological Alliance (2017) found that 53% (*n* = 3459) of the neurology patients they surveyed reported living with at least one other co-morbid condition [7]. Mental health conditions, including anxiety and depression, were among the most frequently reported. For some, a mental health condition can be a clinical symptom of their neurological condition. For others, a mental health condition can be part of coming to terms with diagnosis, the challenges of living with a neurological condition such as maintaining or finding employment, or medication side-effects.

The COVID-19 pandemic has seen many plwLTNCs being placed under shielding restrictions due to their diagnoses and comorbidities [8]. This has resulted in further impact on the mental wellbeing of plwLTNCs. Many stroke survivors have experienced increased social isolation and changes to their mental wellbeing as a result of being unable to leave their homes and as a consequence of restrictions placed on services such as community rehabilitation [9]. The sudden closure of social support services due to COVID has contributed to worse quality of life and anxiety in those affected by dementia across the UK [10]. Young people living with multiple sclerosis, those with a progressive diagnosis or existing psychological symptoms have been found to be more at risk of the negative impact of COVID-19 [11].

The majority of published research exploring ways to improve mental wellbeing has focused predominately on the general population or people living with long-term conditions [12]. The Foresight project [13] aimed to develop a long-term vision for maximising mental capital and wellbeing in the UK and analysed the most important drivers. The report outlined five evidence based actions to improve wellbeing, including connecting with other people; engaging in physical activities; being aware of the world around us, often referred to as mindfulness; trying something new or rediscovering an old interest; and doing something nice for a friend/stranger or volunteering. The report also outlined the impact of external stressors such as debt and poor housing on mental health. In 2018 the UK government published a strategic framework for tackling loneliness. There is substantial evidence to show that loneliness and social isolation are one of the major contributors to ill health in the UK, significantly increasing the risk of premature mortality [14]. Effective interventions to address loneliness include physical activity, befriending schemes, skills training and community based group activities [15].

There is limited research looking at specific interventions or activities to improve wellbeing in people living with LTNC. Research has shown that people living with LTNCs value opportunities for peer support and social interaction [16]. Studies by Ng, Talman and Khan (2011) and Locock and Brown (2010) explored the benefits of peer support groups for people living with motor neurone disease (plwMND) [17, 18]. Peer support provided opportunity to exchange useful information or a sense of camaraderie, while for others witnessing the downward comparison was distressing and accessibility and limited frequency was seen as problematic. The conclusion was that peer support groups were beneficial for some plwMND, but not all. Simpson et al. (2020) identified that the opportunity to engage in a variety of community-based activities was desirable for plwMND and provided purpose [19]. The use of community-based exercise groups has been found to improve the wellbeing of people living with stroke and their family carers [20]. Similarly, Yoga [21, 22] and dancing [23, 24] have been shown to improve wellbeing in addition to physical health for people living with Parkinson’s Disease. The use of mindfulness by people living with Multiple Sclerosis [25, 26] and Traumatic Brain Injury [27] has demonstrated a positive impact on wellbeing. Activities such as gardening [28–30], walking [31], creative tasks [32] and music [33, 34] have been the most widely researched for use with people living with dementia with evidence supporting their use for improving quality of life and wellbeing.

Social prescribing is a means of providing practical support and improving the psychological wellbeing of the population [35]. Social determinates of health are non-medical factors that can positively or negatively influence health outcomes, examples include education, unemployment, housing and social inclusion [36]. Social prescribing aims to tackle these wider social determinants of health [35]. Primary Care Networks formed in 2019 to bring GP practices together to enable them to offer a wider range of services and integrate with the wider health and care system [35]. They have been given funding as part of the NHS long term plan to roll out social prescribing with money allocated to recruit link workers [37]. The NHS England 10 High Impact Actions which outline plans for general practice hopes social prescribing will reduce GP workload and increase capacity [38]. The NHS Long Term Plan outlines plans to develop guidelines for how to promote health and wellbeing within communities, as well supporting the design of local plans to deliver personalised care, which focuses on prevention and wellbeing [39].

Social prescribing has been defined as a formal process of referring people with largely socioeconomic and psychosocial issues to a link worker. Together they co-design a plan to improve the individual’s health and wellbeing [40]. Referrers are most often located within primary care such as GPs, however some social prescribing schemes will accept referrals from other health professionals or even take self-referrals. The link worker facilitates engagement in activities often provided by voluntary and community sector organisations [12]. Examples of social prescribing activities include accessing educational courses, volunteering, attending social clubs, joining in with hobby clubs, dance or art classes [41]. Other forms of support may include accessing debt or housing advice or connecting an individual to employment support services [42, 43].

Research identifying barriers and enablers to implementing social prescribing for long-term conditions such as diabetes, cardiac and respiratory conditions has grown in recent years.. Husk et al. (2019) found that patient motivation, self-efficacy and a belief in the relevance of the activity impacted on enrolment to social prescribing programmes. Cost, transport, venue and timing of the activity impacted on client engagement. Reminder phone calls, written information, introduction sessions or attendance with a ‘buddy’ supported client engagement [44]. Adherence was believed to need trained staff exhibiting good leadership skills; activities that foster interpersonal relationships, trust between link worker and client; supportive environments; and a perceived positive change in their condition with an absence of negative effects. Wildman et al. (2019) explored link workers perceptions of the enablers and barriers to client engagement. Link workers felt they lacked the capacity and/or expertise to offer clients with complex needs the high-intensity and the specialist support they needed. Training focused on the wider determinants of health, behaviour change, mental health issues as well as training on specific long term conditions was seen to be an enabler [45]. These findings were recently echoed by Holding et al. (2020) who found that link workers reported difficulty supporting people living with severe mental health or physical difficulties and identified further barriers related to local infrastructure [46]. Cuts to community organisations funding and the benefits system are believed to be significant barriers to implementing and sustaining social prescribing services [47]. There are no studies examining the barriers and enablers to implementing and engaging with social prescribing from the perspective of people living with LTNCs.

The aim of this study was to understand the experiences of people living with LTNC in engaging with social prescribing services or programmes and the perceived enablers and barriers to participation. Whilst the COVID-19 pandemic has halted nearly all face-to-face social support services, it is important to understand the extent and benefits of social prescribing in a pre- and hopefully soon post-pandemic world. By establishing their understanding, their needs and suggestions for activities to be provided as part of a social prescribing initiative, services can improve adaptation and provide more targeted support for people living with LTNCs.

## Methods

### Participants and recruitment

Convenience sampling was used to recruit patients during the monthly coffee morning at a neurological support charity based in a city in England. The charity provides emotional support, practical help and social activities for plwLTNCs, their family, friends and carers. Various LTNC support groups attend the charity’s coffee morning and were asked to cascade information about the project through their respective groups. People with a diagnosed LTNC and family carers of people with a LTNC (18 years or older) were eligible to participate in the focus groups. Information on how to contact the co-investigator was provided in the patient information sheet, which was given to anyone who expressed an interest in the study. The co-investigator invited participants to a focus group if they met the criteria of the study following discussion via phone or e-mail. Participants were assessed to ensure they had capacity to participate and provided written informed consent prior to participation on the day.

The study was carried out in accordance with the Declaration of Helsinki and received ethical approval from the University of Liverpool Central University Research Ethics Committee B [ID: 5607] prior to study commencement.

### Data and topic guide

A copy of the topic guide can be found in supplementary materials (Additional file 1). Questions were developed by the research team, including a former carer for someone living with MND, and circulated amongst occupational therapy colleagues. The topic guide covered people’s knowledge and experience of social prescribing, their views on social prescribing for plwLTNCs including the potential barriers and enablers to participation. The questions were kept the same for each focus group.

### Procedure

The focus groups were carried out between the December 2019 and February 2020. Groups consisted of a maximum of five participants. Consideration was made in relation to the needs of individuals signing up to each focus group. Groups were kept to a maximum of five participants to enable full participation. Recruitment numbers and enabling choice of dates for groups did not allow for the separation of plwLTNCs and carers in groups and this is acknowledged to be a potential limitation of the study. The co-investigator conducted the focus groups at the neurological support charity. Before the focus groups commenced, the co-investigator assessed the mental capacity of all the people with LTNC and written informed consent was taken. Anyone deemed to lack capacity was excluded from the study. Consent was established through private conversation with the participants prior to the group commencing. Their ability to retain and understand the purposes of the research and their right to withdraw was established. Only one participant had to be excluded as they were unable to give informed consent. The focus groups lasted no longer than 90 min and were audio-recorded. All audio-recordings were subsequently transcribed and anonymised.

### Patient and public involvement

The research team included a former carer of someone living with MND. They provided support and guidance in relation to the design of the project and the study documents, and contributed to the interpretation of findings and dissemination. Reimbursement was provided in accordance with NIHR INVOLVE guidelines [48].

### Data analysis

Focus group data were analysed using inductive thematic analysis. Thematic analysis is a method of exploring, analysing and reporting patterns within themes and identified based on prevalence and/or keyness [49]. Each transcript was analysed by two research team members (CG, SS) individually, by reading through anonymised transcripts repeatedly and highlighting codes pertinent to the research question. Codes were identified in an in inductive process, by not having pre-defined concepts of what overarching themes would emerge. Identified codes were discussed jointly at meetings and themes agreed to merge individual codes which appeared frequently into themes. Table 1 provides an outline of the themes, subthemes and their corresponding codes. Recruitment did not allow confirmation that saturation had been reached.

**Table 1:** Outline of themes, subthemes and their corresponding codes

Theme	Subthemes	Codes
Lack of knowledge	The term social prescribing	Lack of knowledge of social prescribing by plwLTNCs
		Health care professional’s lack of knowledge of social prescribing
	Access to information about LTNCs	Link worker’s knowledge of LTNCs
		Training/education
Service provision difficulties	Knowledgeable well-funded services	Knowledge of LTNCs
		Funding services
		Including plwLTNCs in service design
		Raising awareness of LTNCs
	Bespoke	Variety
		Ability not disability
		One size doesn’t fit all
		Ongoing support
		Cost/finances
Benefits of social prescribing activities	Social isolation	Isolation
		Loss of roles/relationships
		Personal experience
	Social connection	Getting out
		Purpose
		Learning a new skill
		Mental stimulation
		Physical activity/sport
	Belonging	Connection to a group
		Volunteering
		Supporting others
Physical barriers	Accessibility	Environment
		Toilets
		Timings
	Adaptability	Enabling participation
		Adapting activities
		Managing risk
	Driving	Driving
		Public transport
		Taxis
Psychological barriers	Mental health and physical ability	Confidence
		Anxiety
		Physical symptoms
		Family views
	Stigma	Stigma
		Acceptance
		Discrimination
		Other people’s fears/worries

### Reflexivity comment

One research team member (SS) is an occupational therapist and working in the field of long-term neurological conditions, with an MRes degree and some experience in qualitative data. She received further qualitative data support from CG, educated at PhD level in Psychology, who has in-depth experience in conducting qualitative research with vulnerable population groups. Both of their backgrounds made them aware of the population groups as a whole, whilst they were solely guided by the data and not by their background.

### Findings

A total of four focus groups (minimum 4 participants) were carried out with a total of 17 participants, 12 female and 5 male. Participants represented the views of people living with a variety of neurological conditions, this included multiple sclerosis (2), Fragile X Syndrome (1), epilepsy (4), traumatic brain injury (1), essential tremor (2), ataxia (2) and subarachnoid haemorrhage (3). Two participants were family carers and supported people living with epilepsy (1) and motor neurone disease (1).

Across the four focus groups five themes were identified: (1) Lack of knowledge; (2) Service provision difficulties; (3) Benefits of social prescribing activities; (4) Physical barriers and (5) Psychological barriers. Within these themes subthemes were identified and are outlined.

#### Theme 1: lack of knowledge

##### The term social prescribing

The majority of participants had not heard of the term social prescribing. Those who had had been made aware of it by the charity where the focus groups took place or had seen information in the media. Reference was made to the term social prescribing and its link to a medical model of care and the potential difficulties the term would introduce when searching for information.

*“I don’t think I’d necessarily heard the term but I know what that means. I’d seen stuff about people gardening and people being outdoors so I presumed it was all interlinked in to that side of things.”* ***Focus Group 1 Participant***

*“I have heard it in the media and the papers and actually I think there was something on BBC news actually all about it. I might not be right but I think it is all about coming up with something that you can do to aid your condition or your recovery after what might … all of us have some sort of neurological condition so it is about that.”* ***Focus Group 4 Participant***

None of the participants had knowingly accessed a social prescribing service. For the few who had accessed activities or groups, the trigger was a passing comment made by a health professional or was the outcome of their own motivation to participate. Participants highlighted the need for education of health care professionals about social prescribing so that they could consistently signpost or refer people living with LTNCs.

*“ … because it was just like a two-minute thing my consultant recommended coming here because there was no more that he could do, because he was so busy it was just like go there for help with practical support, I hadn’t even thought of it but I was told here (referring to the charity).”* ***Focus Group 2 Participant***

*“I think also it is getting GPs on board as well because if they do, GPs are obviously snowed under aren’t they?”* ***Focus Group 4 Participant***

##### Access to information about LTNCs

Participants felt link workers would need to have knowledge and understanding of LTNCs. There was recognition amongst the groups that this would be a challenge given the number of neurological conditions and the vast array of difficulties faced by people living with LTNCs. Participants made reference to link workers needing good communication and social skills. Participants felt some people living with their conditions would need link workers to use a variety of communication approaches such as e-mail, text message and sykpe as well as face to face to engage with people. For those with memory problems the use of text reminders was highlighted as an important enabler. They felt given the challenge link workers would face, they would need to feel valued and have access to training and support.

*“I think something like this (referring to the charity), or like a booklet for people potentially going in as link workers would help them. Like the different diagnosis, what to expect … ”* ***Focus Group 2 Participant***

*“You need people who are like, no matter who the person is or whether they are autistic or epileptic or I don’t know, no matter what the problem is they’ve got to be able to actually communicate with that person which would probably be difficult to find”* ***Focus Group 3 Participant***

#### Theme 2: service provision difficulties

##### Knowledgeable well-funded services

The need for knowledge from link workers extended to services and activity providers. In order to achieve this understanding, emphasis was placed on the need to involve people living with LTNCs in the development and delivery of services. Participants recognised the cost of providing services and that activities needed to be adequately funded.

*“I mean the big thing is funding isn’t it, I mean, the NHS is struggling and we, you know social prescribing requires money to have a link worker and valuing the fact that this link worker is important. You know you can go to your GP and go and see the specialist nurse and whatever but they don’t fully understand what your condition is and there are so many different conditions so funding is a big thing”* ***Focus Group 4 Participant***

*“It’s all more workers thought isn’t it? You know it’s not an easy fix you know. One to one is all more work and it’s all more expense if it’s being paid for by the NHS.”* ***Focus Group 2 Participant***

##### Bespoke

Recognition of individual differences despite the same diagnosis was important with participants emphasising that one size does not fit all and that activities would need to be varied. Ensuring services got to know the person living with a LTNC and focused on their abilities rather than their disabilities was a priority. Participants considered the impact finances had on the ability to participate in activities as many were unemployed or living on a reduced income. Some highlighted problems negotiating the benefits system.

*“Cause like you were saying before, if your benefit that you’re entitled to is stopped your income is going to go down, so of course money to access anywhere or there being places that are local for you to go to. It’s more expensive to get to if they’re not local anymore”* ***Focus Group 2 Participant***

*“I think with social prescribing for neurological conditions there has to be realisation that not one size fits all, because of the variety of the conditions you can’t just say you’ve got MS, you’ve got epilepsy whatever maybe I’ll send you off to a gardening group. So there has got to be a real thought process behind what’s being prescribed for people.”* ***Focus Group 1 Participant***

*“Maybe it will put a lot of emphasis on the things that you can’t do anymore, and even if you can’t do things anymore, they don’t put the emphasis on all of the stuff that you actually still can do and focus on that instead.”* ***Focus Group 3 Participant***

Participants felt access to support to attend groups would be important and they felt consideration was needed as to how long this support was provided. Participants felt people living with LTNCs may require a more prolonged period of support to be able to continue participation independently. Due to the high levels of anxiety and reduced confidence experienced by plwLTNCs, they believed it was unlikely a single accompanied visit would be sufficient.

*“I think sometimes with neurological conditions the person needs to be there to support them a little bit longer than maybe someone who doesn’t have a neurological condition”* ***Focus Group 4 Participant***

#### Theme 3: benefits of social prescribing activities

##### Social isolation

Participants discussed the difficulties faced by people living with LTNCs. Social isolation was seen as a significant problem in all focus groups. Participants expressed feelings of loss, difficulties maintaining relationships, managing families concerns and the impact of not working.

*“The isolation is the worst part because you go from, well depending on what issue you have, I have gone from what I had which was a very pressurised work environment, kind of working at the top of what I could do in a very busy, I suppose in and I know this sounds stupid, but being quite important in terms of what I did to everything has gone.”* ***Focus Group 1 Participant***

*“I think getting a diagnosis is isolating in itself because you can still have your friends and your family and everyone that you used to have still around you but it’s something that has only happened to you. So things to help people feel less isolated”* ***Focus Group 3 Participant***

##### Social connection

Social prescribing was considered an opportunity to reduce social isolation for people living with LTNCs. Activities provided the opportunity to socialise and connect with others. Attending activities could provide a reason to leave the house and participation in activities provided a sense of purpose. New activities offered the opportunity to learn a new skill and for some this was felt to act as a form of rehabilitation promoting further recovery. Others felt activities provided important mental stimulation. For those participants living with ataxia physical exercise and sport was seen as a valuable activity for supporting mental and physical health as well as providing opportunity to socialise. Participants living brain injury felt participation in activities could aid recovery, whilst others with degenerative conditions focused more on the sense of purpose activities provided.

*“I do a stitch club because my brain has not been working properly and I have been like well if my brain needs to work out new ways to work let’s do something that your brain has never done before, so I stitch and actually from stitching that got me involved in other bits and bobs so it’s just been super cool”* ***Focus Group 1 Participant***

*“I think volunteering gives you a real sense of purpose. It’s really important. Obviously it’s easier to access for people with neurological conditions, looking for a job might be difficult, but getting in to volunteering can be easier”* ***Focus Group 2 Participant***

*“I don’t want to sit at home and not doing anything, I don’t think anyone wants that”* ***Focus Group 4 Participant***

##### Belonging

Participants made particular reference to the benefits of volunteering and peer support. Opportunities to volunteer were key to providing people with feelings of purpose and could provide an alternative to paid employment. Meeting with people with the same or similar conditions was important for many of the participants. Attendees could choose to talk about their conditions and gain support, alternatively they could talk about anything other than their condition, but knew they were with people who understood.

“That’s actually probably the best thing about me being in an environment with people who have got head injuries is that nobody talks to me about it.”

***Focus Group 1 Participant***

#### Theme 4: physical barriers

##### Accessibility

Participants raised a number of concerns regarding accessing social prescribing activities. The availability of activities and the accessibility of buildings was recognised to be a physical barrier to participation. For those participants who were wheelchair users lack of access to an appropriately adapted toilet was often a barrier to participation, some participants explained that they would either not drink or minimise the length of time they were out. Timing of activities was also discussed in relation to how medications or symptoms of a neurological condition can impact on a person’s ability to attend activities at certain times particularly early in the morning. Fatigue was a symptom experienced by the majority of participants and is recognised as a symptom of many neurological conditions.

*“Yes, and even the time of day perhaps as well. I know speaking perhaps for XXX is that she is always better, livelier because of medications in the morning, and we’ve spoken to other people and by the afternoon because of the medications they are on they’re weary and need a rest and tend not to go out in the evenings as well for various reasons, so mornings tend to be, certainly for us and others that we know, are better.”* ***Focus Group 1 Participant***

##### Adaptability

Some participants highlighted the need to consider the activities and the potential need to adapt an activity to enable participation. This was especially pertinent to those who were wheelchair users or had reduced hand function. Risk assessment was also important for those living with epilepsy and having the option to complete activities whilst seated.

*“I have tonic clonic seizures so I could go from kind of like standing up to automatically being on the floor and therefore I am not able to kind of work in a garden, whereas I could quite happily be watching kind of a film … .or discuss books or that sort of thing, it’s a safer environment.”* ***Focus Group 1 Participant***

##### Driving

Diagnosis with a neurological condition can result in temporary or permanent restrictions on driving. All the groups talked about the impact of being unable to drive and issues relating to accessing public or private hire transport.

*“I’m sort of the isolated sort of way because I’m unable to drive due to my condition so when my mum who is also sitting next to me, is out then I can’t drive so I’m sort of isolated. I’m just sort of sat there and there is no outreach like kind of groups for me unless I’m able to get taxis to places”* ***Focus Group 1 Participant***

*“You go out and you might have a seizure on the train or on the train platform or on a bus, and people won’t help you or in a taxi and you might get chucked out the taxi just anywhere or the taxi driver’s going to act real funny about it. So you end up sort of thinking ‘oh, you know, it’s just sensible if I stay at home’ … and then you do stay at home and then you never leave … and then you just get comfortable staying in”* ***Focus Group 3 Participant***

#### Theme 5: psychological barriers

##### Mental health and physical ability

All groups highlighted a number of psychological barriers. Reduced confidence and anxiety was regarded as a barrier to people with LTNCs visiting new places or joining groups and activities. Concerns related to physical symptoms, communication and duration of increasing isolation was discussed. Families’ worries about the person living with a LTNC and needing to manage their concerns was explored as another potential barrier.

*“I’d love to get out and about a bit more but the fear of going out and the fear of tripping that’s what makes me think about, I know I shouldn’t because I’m over reacting or you may think so but me, I’m not. I panic, I am a panicker.”* ***Focus Group 3 Participant***

*“I think one of the biggest issues is the actual carer or family that are trying to maybe cocoon the person with the neurological condition and making assumptions like ‘well I don’t think they can do that’ because they are trying to be overprotective. So I think if the link worker tried to work with the family to ensure the family are and the carers are comfortable because once they feel that comfort then they will work with the link worker”* ***Focus Group 3 Participant***

##### Stigma

Stigma and lack of acceptance by others was seen as a significant barrier for people living with LTNCs. The general public’s awareness of LTNCs was felt to be poor in particular their understanding of hidden or invisible disabilities. Those participants living with epilepsy felt many groups immediately put up barriers when hearing their diagnosis and were worried something might happen to trigger a seizure and the consequences of this for the group. Some participants felt their mental capacity was questioned when they revealed their diagnosis and people would change their behaviour towards them often in a negative way. There appeared to be a strong desire to be accepted and treated like everyone else.

*“Well the main worry is if you haven’t gone so long without a seizure and you’re frightened of people’s reactions... Cause I’ve been stopped by the police for being drunk and I don’t even drink … because they don’t understand the condition, you know there’s people with different disabilities they don’t understand. Because I always say role reversal, you’ve got to have been through it to understand it”* ***Focus Group 3 Participant***

*“I think with anybody who has had a neurological condition physically you look fine, it is what’s going on in the inside and people don’t, don’t know and that is due to lack of knowledge, lack of education.”* ***Focus Group 4 Participant***

*“A lot of people are like ‘oh you can’t do this and you can’t do that’ and you know, you can’t do these things and you’ve got to take these meds and you can’t stay out late at night and you can’t do all these myriad of things where really I probably can or could if I had some support.”* **Focus Group 3 Participant**

## Discussion

These are amongst the first findings to explore the potential benefits and perceived barriers to accessing social prescribing for people living with LTNCs. Participants highlighted that there was very little knowledge of social prescribing amongst people living with LTNCs. Those who had accessed social prescribing activities had done so by chance and as a result of their own proactiveness. The lack of signposting by health professionals was apparent and there was an agreed need for health professionals to be educated about social prescribing. Bickerdike et al. (2017) reported that social prescribing was unfamiliar to many GPs and in order to engage participants they required a good clear explanation [50]. This sentiment is echoed by Bertotti et al. (2018) who found that ‘buy in’ from GPs was essential with adequate time allocated during consultations to explain social prescribing [51]. The findings of this study suggest GPs and health professionals are not consistently referring people living with LTNCs to social prescribing and the reasons for this should be explored.

The benefits of participation in activities was recognised by all the focus groups. Participation was seen to provide meaning and purpose. A strong emphasis was placed on the social benefits of participation. Similar findings have been demonstrated in social prescribing studies. An evaluation of a social prescribing service in Northern England found that accessing a range of activities provided a sense of independence and gave individuals a sense of purpose [52]. A recent study reported that the social prescribing pathway facilitates group membership providing valuable social connection, this was found to improve quality of life for people living with long term health conditions [53]. Social isolation and loneliness is experienced by many people living with a neurological condition. It is well documented that, in the long-term, individuals with traumatic brain injury (TBI) are less active in social and leisure activities and they experience a drastic decrease in the number of friends and the frequency of social contact [54]. The same can be said for those people living with dementia especially if living alone [55].

Stigma was found to impact on social identity leading to loss of confidence and anxiety. Research into stigma particularly the impact of living with epilepsy has shown that stigma can result in a perceived reduction in social value and poor quality of life [56]. Interventions to address stigma with people affected by HIV [57] and mental health conditions [58] has been widely published and emphasise the importance of social connection and education. There is little evidence to guide interventions to address stigma with plwLTNCs and is an area that needs greater research attention. Peer support was an important element of social participation as this helped to provide a sense of acceptance and belonging. Research suggests peer support helps people to manage their long term conditions and should be valued [59]. Participants in this study did not voice any reservations about mixing with other plwLTNCs. This differs to the findings highlighted earlier examining MND peer support groups and that some participants found seeing others in the later stages of MND distressing. This could be a concern reserved for this patient group given the rapidly deteriorating nature of MND and not applicable to all plwLTNCs. Having access to support to attend activities was important to aid confidence. Siette et al. (2017) suggest that befriending interventions can potentially influence mental health outcomes and personal relationships for people living with mental and physical health problems [60].

Support to return to work or to find volunteering opportunities was a desirable area for link worker support for many of the participants. People with LTNCs who fail to return to work after injury or onset, or who are encouraged to relinquish work prematurely may be financially disadvantaged, have a poorer quality of life and suffer adverse health outcomes such as anxiety and depression [61–63]. For example, returning to work or education is a major goal for many people who sustain a TBI but only about 41% are in work at one and 2 years post injury [64]. Studies of employment and work loss in Multiple Sclerosis cite unemployment rates ranging from 24 to 80% and unemployment has been associated with disease progression and an increase in symptoms [65]. These consequences result in increased consumption of health resources including GP services and consultant contacts. Volunteering has been found to provide a sense of belonging and positively influence wellbeing [66]. A study by George and Singer (2011) found that volunteering in a school and supporting children aged 5–14 with their education reduced stress levels in people living with mild and moderate dementia [67]. Providing support and opportunities for volunteering could have a positive impact on plwLTNCs mental wellbeing and should be promoted.

Participants discussed the physical barriers to accessing social prescribing activities. Transport was seen as a significant barrier to participation. Many studies have highlighted that transportation is often a barrier to participation in community based activities [19, 68–70]. Transport was found to impact on the likelihood of attendance when examining what works for who and in what circumstances in social prescribing [44]. The lack of fully accessible toilets with changing facilities is recognised as a barrier to community access for people living with cerebral palsy, MS, MND, head injuries, severe spinal injuries and stroke [71]. Without changes to local infrastructure, transport and accessibility will continue to be a barrier for many people.

Participants emphasised the need for knowledgeable link workers and services given the complexity of LTNCs, the challenges faced by many living with a neurological condition and the importance of individuality. A recent systematic review [16] examined the experiences of people living with LTNCs engagement with community rehabilitation and support services. They found that outcomes of self-efficacy and self-management were important for people with stable and progressive LTNCs. Interactions with individual professionals were found to influence engagement and desired outcomes. As a result, training should develop the advanced communication skills and behaviours required to facilitate self-efficacy and self-management.

### Recommendations for practice

The provision of group and individual programmes that promote health, social interaction and self-efficacy have been at the forefront of occupational therapy intervention historically [72]. According to the Royal College of Occupational Therapy (RCOT) (2019) we are seeing a cultural shift in the way health and care is perceived and delivered, placing what matters to the individual at the heart [73]. Occupational therapy’s primary goal is to enable people to engage in their chosen activities, this is achieved by maximising their ability to participate, or by adapting the activity or environment to enable engagement [74]. Occupational therapy has a lot to offer in delivering personalised care. They have a unique contribution to make to social prescribing one that utilises their expertise effectively, however they are not an infinite resource and should be used wisely [73].

Occupational therapists should be recruited to work alongside link workers to reach those living with LTNCs in order to tackle many of the barriers highlighted in this study. With the push to introduce New Roles in primary care networks this could be achieved through direct referral to occupational therapists based within primary care who would then work collaboratively with a link worker [74]. This model has recently been supported by the Royal College of Psychiatrists who emphasised the need for a clear pathway to occupational therapist’s expertise when a person’s needs go beyond the level of competence and training of a link worker [75]. A study by Simpson et al. (2020) found that link workers valued having access to an occupational therapist when supporting plwMND due to the complexity of the condition and the barriers they faced [19]. Reablement services have successfully implemented this type of service model. Reablement is a person centred approach which aims to promote faster recovery from illness, prevent unnecessary admissions to hospital and increase a person’s independence in their own home. Evidence from practice and research suggests that the most successful reablement teams have occupational therapy input [76]. There are already successful examples of occupational therapy lead social prescribing services such as the Ways to Wellbeing Service based in York [73].

The National Institute for Health and Care Excellence (NICE) public health guideline ‘Mental Wellbeing in over 65’s’ [77] focuses on enabling older adults to participate in meaningful, physical and social activity in order to prevent mental and physical ill health associated with factors such as social isolation and discrimination. The problems experienced by older adults echo many of those experienced by plwLTNCs. The guideline recommends the involvement of occupational therapists in the design and development of locally relevant training schemes for staff supporting older adults. Training schemes should provide essential knowledge of (and application of) the principles and methods of occupational therapy and health and wellbeing promotion, as well as effective communication skills. Occupational Therapists could therefore be the most well equipped profession to supervise and train link workers. In 2019 The Royal Society for Public Health, NHS Improvement, NHS England and Public Health England developed the Allied Health Professionals (AHP) social prescribing framework. The framework covers the range of AHP engagement in social prescribing and highlights the important role occupational therapists can play in the development and delivery of social prescribing services [78].

### Limitations

This study was subject to some limitations. It is recognised that the study included a small number of plwLTNC and participants and may not be representative of all LTNCs. As a result of convenience sampling, our study included people with multiple sclerosis, Fragile X Syndrome, epilepsy, traumatic brain injury, essential tremor, ataxia and subarachnoid haemorrhage. The family carers supported people living with epilepsy and motor neurone disease. The investigators took the decision to keep demographic data to a minimum in order to help participants to feel their responses would be completely anonymised especially given the small sample size. This article will be accessible to all and that will include the charity where the study took place. Details regarding age, ethnicity and locality were not collected and quotes do not indicate who is responding or their diagnosis as this would identify who the respondent was especially single representatives of a condition. However, this is one of the very first studies exploring social prescribing with plwLTNCs, indicating steps for future research to build on, such as expanding the participant pool to incorporate a greater variation of LTNCs. The majority of participants were regular attendees at the charity where the focus groups were run and this may have influenced their responses leading to potential response bias. This might have included avoiding making critical comments about the activities run by the charity or adapting responses due to being familiar with other members of the focus group. The decision to have mixed groups of family carers and plwLTNCs is acknowledged to be another limitation of the study as the presence of each might have resulted in people holding back to avoid offending or upsetting another participant due to their status. The study may not represent those plwLTNC who experience significant difficulty leaving the house and are in greater need of social prescribing than those people represented in this study. Future research should aim to reach out to those who are unable to leave their homes to establish if they experience the same or different barriers to social prescribing activities. Research should also examine the impact the pandemic has had on the wellbeing of plwLTNCS. This should include potential positive changes such as online groups and activities which aimed to enable social interaction and inclusion during the pandemic [79], but also consider the barriers to accessing activities remotely and actions needed to prevent further inequalities [80]. Research should consider how technology might be utilised in social prescribing whilst balancing this with opportunities for social connection face to face.

## Conclusions

This study emphasises the need to consider the whole system and how social prescribing can be framed to meet the needs of plwLTNC. Future research should explore how best to enable participation by overcoming physical and psychological barriers and by identifying interventions that reduce the impact of psychological barriers such as stigma. A particular focus needs to be set on the impact of the COVID-19 pandemic on engaging with social prescribing, as this is likely to have thrown up further barriers in accessing general social support, as emerging evidence highlights. The pandemic has also provided positive learning opportunities and the potential to utilise technology in the delivery of social prescribing going forward. Local infrastructure needs to evolve in order to reduce the physical barriers faced by plwLTNC in particular transport. Without major changes, plwLTNC will endure further inequalities relating to the delivery and accessibility of social prescribing. This paper has made recommendations to consider the role occupational therapists could take in the development and delivery of social prescribing given their expertise.

## Supplementary Information

**Additional file 1.** Focus group topic guide.

## Data Availability

The datasets used and/or analysed during the current study are available from the corresponding author on reasonable request.
