# Supplementary material for: Exploring the enablers and barriers to social prescribing for people living with long-term neurological conditions: a focus group investigation
Source: BMC Health Serv Res. 2021 Nov 13;21:1230. doi: 10.1186/s12913-021-07213-6 (PMC8590354; doi:10.1186/s12913-021-07213-6)
Supplement: Supplementary file 1 — Additional file 1. Focus group topic guide. [file 12913_2021_7213_MOESM1_ESM.docx]

Focus Groups LTNC and Social Prescribing

Interview Schedule

1. Have you heard of social prescribing and if so what do you understand it to be?
2. Has anyone had any experience of being offered social prescribing and if so can you tell us about your experience?

Social Prescribing is an initiative being supported by the NHS and GPs to help people to improve their wellbeing and quality of life.

GPs or other health professionals refer an individual to a link worker who supports them to identify issues that are impacting on their wellbeing such as loneliness, money worries or poor lifestyle behaviours such as smoking.

The link worker supports the individual to access services that provide practical support with issues such as debts. They will support people to access local community based activities such as art groups, gardening groups and exercise classes to help reduce loneliness. They may also support people to explore courses, volunteering opportunities and employment when appropriate.

1. Having now been you all told more about social prescribing, how do you think it might apply to people living with neurological conditions? Prompt: What types of problems do you experience that social prescribing might help.
2. Thinking about the link workers role in helping people access local community based activities what activities do you think people living with neurological conditions would want to access and why? Prompt: What do you think would be most popular.
3. What do you think would be the biggest difficulties or barriers to people with neurological conditions to accessing social prescribing? Prompt: If a link worker was to work with you what difficulties might they encounter when trying to support you to access activities.
4. What actions or changes would enable someone with a neurological condition to benefit from social prescribing? Prompt: This could be at a local or government level.
